# Analysis of Mental Workload in Online Shopping: Are Augmented and Virtual Reality Consistent?

**DOI:** 10.3389/fpsyg.2017.00071

**Published:** 2017-01-26

**Authors:** Xiaojun Zhao, Changxiu Shi, Xuqun You, Chenming Zong

**Affiliations:** ^1^School of Education, Hebei UniversityBaoding, China; ^2^School of Criminal Justice, China University of Political Science and LawBeijing, China; ^3^School of Psychology, Shaanxi Normal UniversityXi’an, China

**Keywords:** mental workload, online shopping, user experience, virtual reality, augmented reality

## Abstract

A market research company (Nielsen) reported that consumers in the Asia-Pacific region have become the most active group in online shopping. Focusing on augmented reality (AR), which is one of three major techniques used to change the method of shopping in the future, this study used a mixed design to discuss the influences of the method of online shopping, user gender, cognitive style, product value, and sensory channel on mental workload in virtual reality (VR) and AR situations. The results showed that males’ mental workloads were significantly higher than females’. For males, high-value products’ mental workload was significantly higher than that of low-value products. In the VR situation, the visual mental workload of field-independent and field-dependent consumers showed a significant difference, but the difference was reduced under audio–visual conditions. In the AR situation, the visual mental workload of field-independent and field-dependent consumers showed a significant difference, but the difference increased under audio–visual conditions. This study provided a psychological study of online shopping with AR and VR technology with applications in the future. Based on the perspective of embodied cognition, AR online shopping may be potential focus of research and market application. For the future design of online shopping platforms and the updating of user experience, this study provides a reference.

## Introduction

The use of virtual reality (VR) technology and augmented reality (AR) technology in network marketing practices is on the rise. Especially, a market research company (Nielsen) reported that consumers in the Asia-Pacific region have become the most active group in online shopping. Online shopping also represented the fourth retail revolution. AR technology is one of three major technologies identified to be likely to change the future of shopping (the others are QR codes and mobile payment). Based on the perspective of embodied cognition, AR online shopping may be potential focus of research and market application. However, studies based on the technology were less relevant with psychological phenomena. Under the condition of VR and AR, this study aims to investigate the difference of mental workload. For the future design of online shopping platforms and the updating of user experience, this study provides a reference.

### From Virtual Reality to Augmented Reality

Virtual reality technology represents a model of the real world produced by a computer (Kist, 1996). AR technology is an important branch of VR technology. AR refers to a wide variety of hardware technology used to create an annotated composite based on real-world scenarios. AR is used to make a video display of real situations and computer-generated virtual content to undergo real-time and synchronous fusion (Mullen, 2013). Improving participants’ perceptions of the real world is one of the primary advantages of AR (Azuma et al., 2001). Educational psychology, psychotherapy, interpersonal relations psychology, and human factor engineering psychology are fields of study that have played an integral part in the testing of AR technology.

### Business Development and Applied Psychological Research Based on AR Technology

Through both social mechanisms (via community identification) and psychological mechanisms (via trust and satisfaction), virtual community participation has greatly promoted loyalty intentions (Pei-Yu and Hsien-Tung, 2011). Users’ mental workload and situation awareness (SA) are the keys to the supervisory control of safety-critical systems (Yang et al., 2012).

The Vuzix company developed Vuzix Star 1200 to enhance the glasses of the average consumer. This technology can fuse real world images and intelligent mobile phone information. Recently, Microsoft developed an AR projector. This technology uses 4 Kinect to realize the aim of interactions between participants and the image. Google is improving AR glasses, in which the scenery will stack corresponding intelligent information. IBMLabs stated that they developed a mobile shopping application prototype based on AR technology. The Mattel and Fox Films sales department launched a joint series of Avatar toys with AR. HoloLens, made by Microsoft, represents the industry’s confidence in the development of AR. The preliminary AR application of Taobao and Amazon can portray AR as a new technology into the field of consumption. To accelerate the development of technology, relevant research on internal psychological mechanisms is needed.

### The Mental Workload of Online Shopping Based on VR or AR

Early research on the mental workload can be traced back to the study of Knowles in 1960s (Knowles, 1963). The study mainly involved subtask and subjective evaluation technology. It first formed the concept of mental workload produced from a special academic conference on the theory and measurement of mental workload in 1977. The North Atlantic Treaty Organization held a meeting and concluded that mental workload is the synthesis of the job requirement, time requirement, the employee’s ability, degree of effort, and behavior, and other factors. This was a multidimensional concept. The early studies of some experts on mental workload continued this comprehensive analysis. The latest studies showed that the mental workload measuring technique was mainly divided into psychological subjective evaluation technology, physical measurement technology and task measurement technology. The psychological subjective evaluation technology became a mainstream method of analysis. The main tools of psychological subjective evaluation technology included the NASA Task Load Index (TLX), the fuzzy linguistic multi-criteria method, the Multiple Resources Questionnaire (MRQ), the Dundee Stress State Questionnaire (DSSQ), and Subjective Cognitive Load (SCL). This technology adopted the Likert scale for psychological measurement (Liou and Wang, 1994; Wilson, 1998; Wiebe et al., 2010; Felton et al., 2012; Klein et al., 2012). The main tools of physical measurement technology included electroencephalogram (EEG), event-related potentials (ERPs), and eye movement (May et al., 1990; Kramer et al., 1995; Noel et al., 2005; Ryu and Myung, 2005; Evstigneev et al., 2008; Miller et al., 2011; Tokuda et al., 2011; Di Stasi et al., 2013; Borghini et al., 2014). The task measurement technology included real-situation tasks and visual-situation tasks (Törnros and Bolling, 2006; de Waard et al., 2008; Cantin et al., 2009; Jou et al., 2009; Hoogendoorn et al., 2010).

The “Flow” system from Amazon combines online shopping and AR. To reduce the mental workload, the role of AR technology based on experience marketing characteristics should not be ignored. Mental workload was one of the important themes for human factor engineering and the man-machine design field. To achieve job satisfaction, the control of job enrichment and mental workload are necessary (Cook and Salvendy, 1999). In modern psychology, the mental workload is not only efficient but also related to safety and mental health problems. In the VR environment, these factors are established through modeling techniques. Due to the lack of direct involvement, the individual actually increases contrast activity between the model and self. From the perspective of AR, psychological research on online shopping helps shoppers to perceive commodity information more accurately, reduce the time investment, and attract the eyes of consumers. The formation of embodied cognition is through the body’s experience and its activity. The cognition (such as mental workload) of AR online shopping is through the body’s online experience and self. Mental demand, physical demand, temporal demand, performance, effort, and frustration were the six main factors of mental workload. These factors influenced the effect and quality of online shopping. Therefore, the study of mental workload in online shopping is of great significance.

## The Current Study

Under the condition of VR and AR, this study aims to investigate the difference of mental workload. For the future design of online shopping platforms and the updating of user experience, this study provides a reference. The use of VR and AR technology in network marketing practices is on the rise. However, studies based on the technology were less relevant with psychological phenomena. Based on the AR technology, from the perspective of the user experience of online shopping, the study tested the mental workload about VR and AR. The study investigated the differences in the users’ gender and cognitive style, product value, and sensory channel in the VR situation and the AR situation. From the perspective of AR, psychological research on online shopping helps shoppers to perceive commodity information more accurately, reduce the time investment, and attract the eyes of consumers. Males would not be too keen on price factors, paying more attention to the product itself and the efficiency of the shopping experience (Zhang and Zhang, 2012). A high value may cause the user to take on more responsibility. Previous studies have found that personality traits have become one of the powerful predictors of performance, and personality traits can lead to problems with mental workload (Sohn and Jo, 2003; Yoon et al., 2015). At the same time, the cognitive method is one of many personality factors, and cognitive style and mental load influence each other, ultimately affecting the decision-making behavior (Kanter and Coury, 1984). Field-independent users are not affected by the shopping environment. However, field dependence is easily affected by the shopping environment. Based on the above analysis, the study formulated the following hypotheses:

H1 The property of high value is the main influencing factor of high mental workload for males.
H2 In the VR situation, mental workload of users decrease for field dependent when perceptual information is added through sound.
H3 In the AR situation, mental workload of users increase for field dependent when perceptual information is added through sound.

## Materials and Methods

### Aims of the Research

Focusing on the AR, which is one of three major techniques used to change the method of shopping in the future, this study used a mixed design to discuss the influences of the method of online shopping, user gender, cognitive style, product value, and the sensory channel on mental workload in VR and AR situations.

### Participants

Thirty-six participants who intended to purchase furniture or visited a furniture exhibition hall (22 females, *M*_age_ = 29.81, *SD*_age_ = 2.61, 16 field-independent) participated in the experiment. All the participants were right-handed, had normal or corrected-to-normal vision and no color blindness or color weakness, and had no similar experimental experiences before. First, the experimenter randomly selected shoppers near the exit of the furniture store as the possible participants. The experimenter asked the participants the following two questions: (1) Have you ever shopped online? (2) Are you willing to participate in an online shopping experiment for a sofa and mirror? The researcher recruited those who agreed as formal participants. Second, this study used snowball sampling technology by asking the initial participants to recruit related individuals to participate in the experiment. After being recruited, the participants were asked finished their task in their own homes (area is 25 to 35 m^2^).

### Instruments and Tools

#### IPad 2

An iPad 2 with iOS5 and a front and rear camera, speaker, multi-touch IPS fingerprint resistance, a 1024×768 resolution screen, an oil-resistant coating, and 132 PPI pixel densities was used. Its induction module included a delight induction module (to sense ambient light conditions and inform the processing chip to adjust the display brightness automatically; an ambient light sensor needs an infrared cut-off membrane on the chip surface and an even plating hypothetical infrared cut-off membrane on the silicon wafer), the acceleration induction module, and the triaxial gyro, which could simultaneously determinate six directions, positions, movements, and accelerations. In addition, the iPad was equipped with GPS, cellular data, gravity sensors, and a distance sensor.

#### SnapShop Software

**FIGURE 1:**
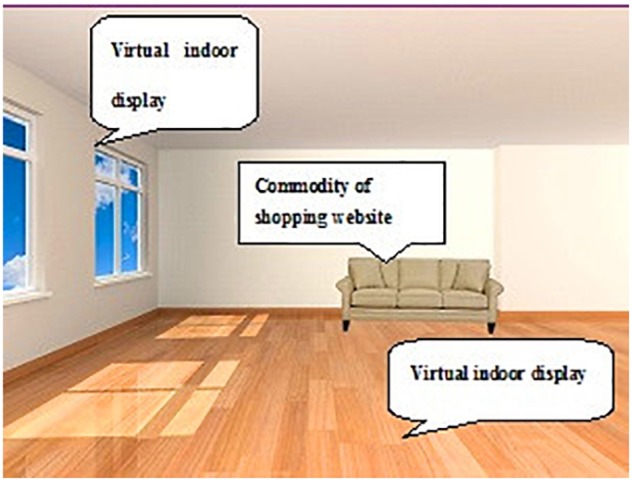
**Online shopping rendering of VR**.

**FIGURE 2:**
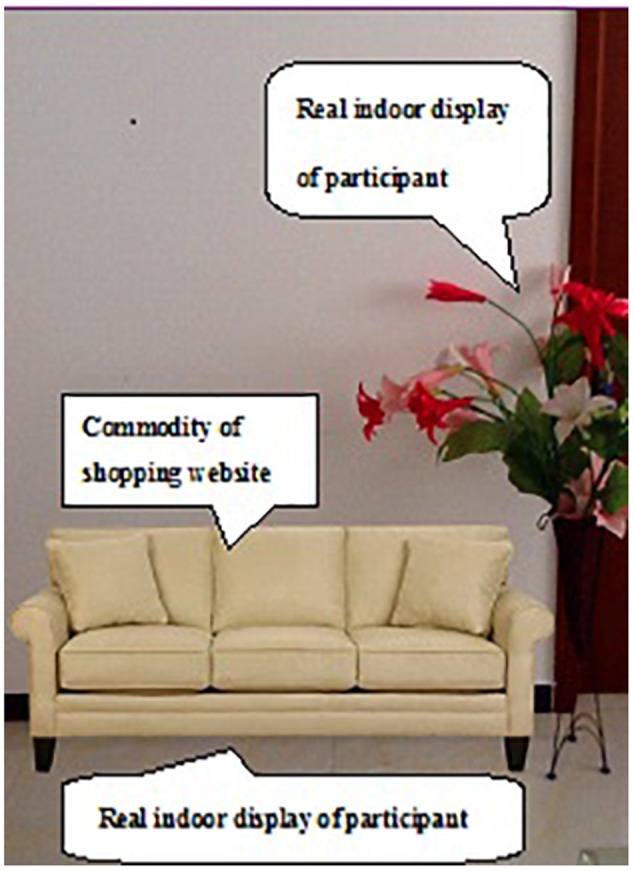
**Online shopping rendering of AR**.

SnapShop Showroom (software developed by SnapShop Inc.) for the iPad 2 determined whether the furniture was decorated in the rendering. After choosing a product, the product image floated on the camera and could be added to the interior space. The simulated rendering could also be saved and shared with friends. This experiment used a virtual background room that belonged to the software and an authentic depiction of the house for participants. **Figure 1** shows a VR online shopping rendering. Using the iPad 2, the user viewed a stack effect between virtual products and indoor facilities using a real-time viewfinder of SnapShop software, ultimately achieving the goal of AR (see **Figure 2**).

#### Downsizing Recording Software

Downsizing recording software compatible with the iPad 2 could easily record, export, and broadcast data. The experiment was conducted in the audio–visual integration channel condition. The participants were asked to view IKEA products in the network advertisement and listen to an IKEA advertisement, which was produced by downsizing the recording software (male voice) at the same time.

#### Embedded Figure Test

The embedded figure test (EFT) was the measurement used to test cognitive style (field dependence or field independence), which was revised by the Department of Psychology, Beijing Normal University (Xie and Zhang, 1988). In this test, the participants were asked to identify a simple figure embedded in the complex. If they found it as soon as possible, their cognitive style was defined as field-independent; otherwise, it was field-dependent.

#### NASA-TLX Scale

This self-reported scale (Hart and Staveland, 1998) required participants to score themselves by the Richter scale for mental demand, physical demand, temporal demand, performance, effort, and frustration in those six dimensions. Then, it required them to set the weight of each dimension. According to the significance ranks of each dimension, we obtained the mental workload. In the test, the internal consistency coefficient (α coefficient) of the scale was 0.737.

### Design

This experiment used a 2 (online shopping method: VR vs. AR) × 2 (user gender: male vs. female) × 2 (user cognitive style: field dependence vs. field independence) × 2 (product value: high vs. low) × 2 (sensory channel: visual vs. audio–visual integration) mixed design. The online shopping method, product value, and sensory channel were independent variables, and the mental workload was the dependent variable.

### Procedure

The experimenter entered the subject’s apartment, conducting a mental workload experiment for online shopping after a brief conversation. The experiment was conducted in the sitting room. Because of the high homogeneity of customers in the market, the areas of their sitting room ranged between 25 and 35 m^2^.

First, participants completed the informed consent form and basic information form. Second, participants conducted a cognitive style test by EFT. Third, the items were prepared. The iPad 2 was connected to the Internet (WLAN). The experimenter demoed the online shopping process using APP (SnapShop Showroom software) to participants.

Fourth, the experiment conducted a psychological experiment of online shopping. There were eight experimental treatments for each participant, including the following: VR – low value – visual conditions, VR – low value – audio and visual integration conditions, VR – high value – visual conditions, VR – high value – audio and visual integration conditions, AR – low value – visual conditions, AR – low value – audio and visual integration conditions, AR – high value – visual conditions, AR – high value – audio and visual integration conditions. In the experiment, four experimental treatments for VR (randomized experiment order) were conducted by participants; then, four experimental treatments for AR (randomized experiment order) were conducted by participants. The participants themselves entered the main page of APP and were classified. In the visual experiment, the model of product chosen by participants and the software’s own indoor scene model could be added together. By operating the touch screen, participants could move the furniture to any position. After choosing it, if the participant decided to purchase it, they placed it in their shopping cart; otherwise, they gave up. In the AR experiment, the model of product choosing by participants and the authentic scene of the house could be added by the cameras of the iPad 2 to combine the virtual and real scenes. By operating the touch screen, participants could move the furniture to any position. After choosing it, if the participant decided to purchase the furniture (with a price tag), they placed it in their shopping cart; otherwise, they gave up. Study participants were recruited from visitors to a furniture store. The experimenter compared the price of a sofa and mirror in the store. In this experiment, the sofa was defined as a high-value product; the mirror was defined as a low-value product. The visual shopping interface was produced from SnapShop Showroom software. Audio advertisement was broadcasted by downsizing recording software (commercial advertisement from Ikea). In audio–visual integration, the experiment synchronized the visual interface and recording auditory advertisement (loop). Participants could store or share photos added together with the model of the product chosen by participants and the authentic scene of the house. Under the eight experimental treatments, all the participants were tested by NASA-TLX.

## Results

**FIGURE 3:**
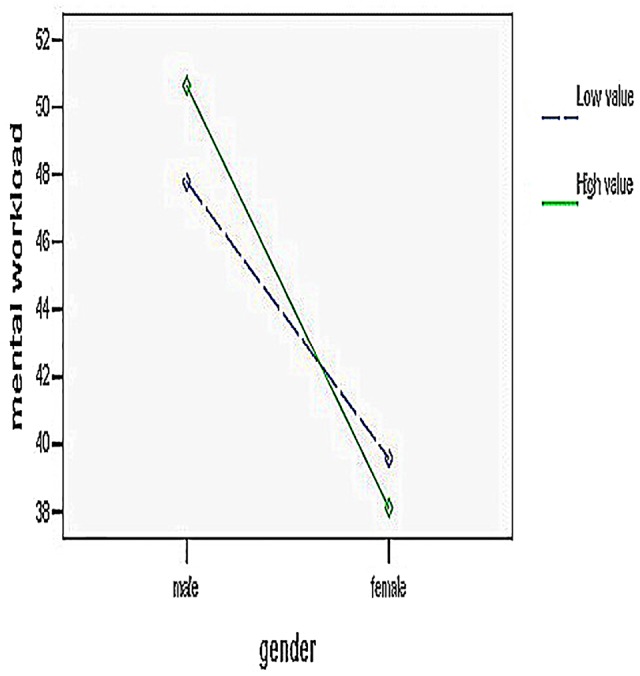
**The interaction between gender and value**.

**FIGURE 4:**
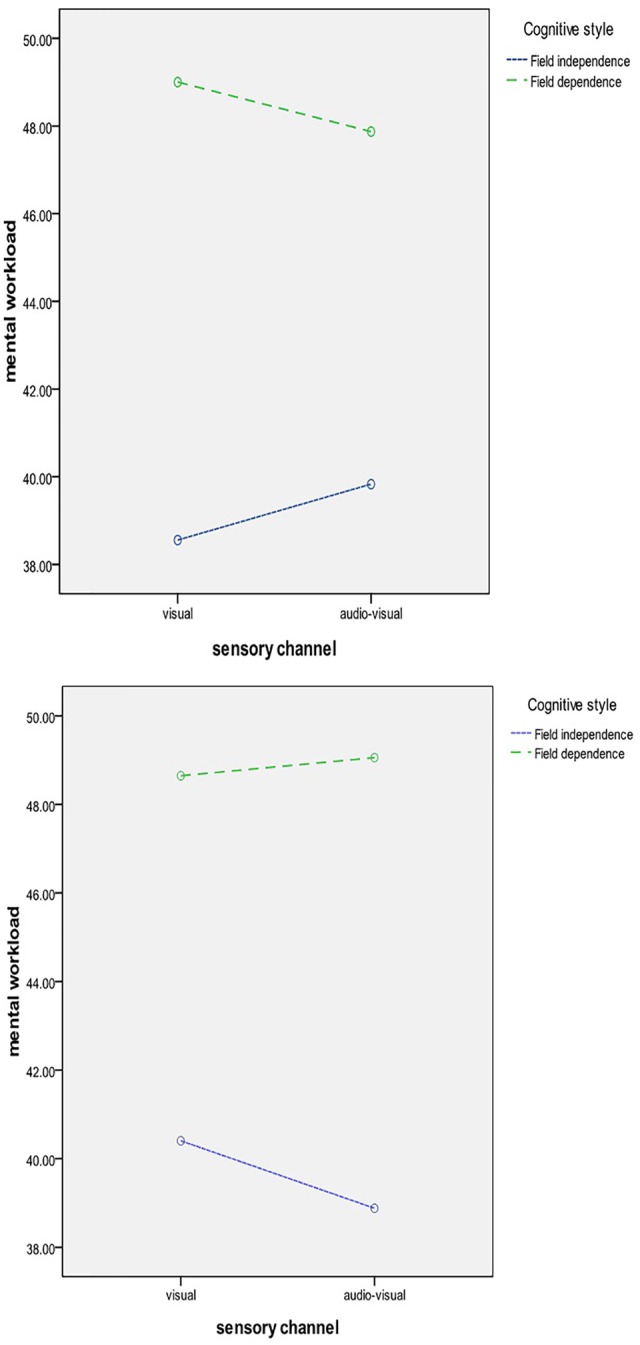
**The interaction between the online shopping method, channel, and cognitive style**.

Researchers performed data management using SPSS 17.0 software. The study used ANOVA, multiple comparisons and analysis of simple effects. The repeated-measures ANOVA revealed that the main effects of gender were significant [*F*(1,32) = 4.20, *p* < 0.05, $\eta_{p}^{2}$ = 0.12]. The main effects of online shopping method were not significant [*F*(1,32) = 0.44, *p* > 0.05]. The main effects of product value [*F*(1,32) = 0.63, *p* > 0.05], sensory channel [*F*(1,32) = 0.24, *p* > 0.05], and cognitive style were not significant [*F*(1,32) = 3.33, *p* > 0.05]. The interaction between the user gender and product value was significant [*F*(1,32) = 6.08, *p* < 0.05, $\eta_{p}^{2}$ = 0.16]. The interaction between the online shopping method, sensory channel, and cognitive style was also significant [*F*(1,32) = 4.22, *p* < 0.05, $\eta_{p}^{2}$ = 0.12]. The remaining interactions were not significant. Combined with the results of multiple comparisons analysis, males’ mental workload was significantly higher than females’ (49.21 vs. 37.94). The simple effects analyses (see **Figure 3**) showed that for the males, high-value products’ mental workload was significantly higher than that of low-value products (50.64 vs. 47.78, *p* < 0.05). H1 is confirmed. In the VR situation (see **Figure 4**), the visual mental workload of field-independent and field-dependent consumer showed a significant difference (38.56 vs. 49.00), but the difference was in reducing in audio–visual condition (39.83 vs. 47.87). Mental workload of users for field dependent was difference in visual and audio–visual conditions (49.00 vs. 47.87). H2 is confirmed. In the AR situation, the visual mental workload of field-independent and field-dependent consumers showed a significant difference (40.40 vs. 48.65), but the difference was an increase in audio–visual conditions (38.88 vs. 49.46). Mental workload of users for field dependent was difference in visual and audio–visual conditions (48.65 vs. 49.46). H3 is confirmed.

## Discussion

### Variance Analysis of Online Shopping in VR

Virtual reality technology represents a model of the real world produced by a computer (Kist, 1996). In the VR situation, males’ mental workload was greater than females’ mental workload. Although the study adopted VR technology, this kind of platform and traditional online shopping were still similar. In online shopping, males were more focused on the practical characteristics of the product and the intervention of rational thinking. Females were more focused on the entertainment and emotional catharsis. Therefore, the males’ mental workload was greater. Zhang concluded that the numbers of female online shoppers were greater than males. Females consider that shopping more fun and enjoyed price factors. Males were not too keen on price factors and paid more attention to the product itself and the efficiency of the shopping (Zhang and Zhang, 2012). Generally, in online shopping, the mental workload was also different by gender.

In the VR situation, the visual mental workload of field-independent and field-dependent consumers showed a significant difference, but the difference was in the reduction of audio–visual conditions. Field-dependent consumers relied more on the surrounding environment as a frame of reference. Because of the effect of the cognitive information on visual advertising, the mental workload of field-dependent consumers was increasingly affected by the visual information. However, if the visual information added auditory content, consumers would have a more comprehensive knowledge of products, and this situation reduced mental workload. Field-independent consumers relied more on themselves as a frame of reference. The audio–visual integration information increased their mental workload.

### Variance Analysis of Online Shopping in AR

Augmented reality technology is one of three major technologies identified as likely to change the future of shopping (the others are QR codes and mobile payment). In the AR situation, males’ mental workload was greater than females’ mental workload. Considering that males shop online less frequently than females (Zhang and Zhang, 2012), they would be cautious when deciding whether or not to buy a product. Additionally, for the AR technique used in this experiment, the males experienced a close relationship between products and themselves. Therefore, they exerted more mental energy into the purchasing process, and their mental workload increased. The property of high value was a main influencing factor of high mental workload for males. In the AR situation, males experienced a higher mental workload when they decided whether to buy an expensive product. In general, males did not shop frequently, but when they chose items, the price was higher, and the quality was better. Males focused on the product itself (Zhang and Zhang, 2012). Males paid more attention to practical characteristics, such as the service time of high-value products; therefore, the mental workload increased.

In this experiment, participants combined the product with their own home because the AR combined the real situation and virtual situation. The AR technology adds computer technology to promote human–computer interactions to enhance the user’s perception toward the realistic world (Azuma et al., 2001). This kind of combination made the buyer more deeply involved in the shopping experience. In the AR situation, the visual mental workload of field-independent and field-dependent consumers was significantly different, but the difference was an increase in the audio–visual condition. The field independence and field dependence influenced the buyers’ degree of involvement, which should be encouraged in AR situations because AR technology itself is an embodiment of the real world.

Field-dependent consumers relied more on the surrounding environment as a frame of reference. Information perception has become an important factor in AR shopping (Rese et al., 2014). Because of the effect of real-world information, the mental workload of field-dependent consumers was higher. If the information included auditory content, consumers were focused on the real information of the room, which increased mental workload. Field-independent consumers relied more on themselves as a frame of reference. Audio–visual integration information was integrated with virtual and real scenes. For those field-independent individuals, the self-concept ruled this kind of involvement in the self-image and self-object shopping process. External stimulant characteristics of advertising did not influence the purchasing behavior. However, this situation could help consumers obtain product information. In addition, pleasure perception became an important factor of AR shopping (Rese et al., 2014) for field-independent consumers. Audio–visual integration information and pleasure perception decreased their mental workload.

### Are Augmented and Virtual Reality Consistent?

The user’s mental workload is different for AR and VR. The sensory channel and cognitive style have become the main factors for inconsistency. Firstly, field dependence is easily affected by the shopping environment. In the VR environment, multiple modalities (vision and auditory) decrease the mental workload for field-dependent users because most of the senses are constructed by themselves, not shopping environment. The construction of feeling is accelerating cognitive process. In the AR environment, multiple modalities increase mental workload for field-dependent users because most of the senses are generated based on the real world. The machining process consumes a lot of cognitive resources. However, field-independent users are not affected by the shopping environment. Secondly, the formation of embodied cognition is through the body’s experience and its activity. The cognition (such as mental workload) of AR online shopping is through the body’s online experience and self. The cognition of AR online shopping essentially reflects the characteristics of embodied cognition. In fact, the relationship of embodied cognition and social consumption has become the important research area (Lee et al., 2014). AR is used to make a video display of real situations and computer-generated virtual content to undergo real-time and synchronous fusion (Mullen, 2013). Improving participants’ perceptions of the real world is one of the primary advantages of AR (Azuma et al., 2001). Field dependence is easily affected by the shopping environment. So, in AR online shopping, AR itself is a kind of environment setting. Fidelity of the visual and auditory environment become an important source of mental workload for field dependence. Therefore, background data collection of user should be an immediate response to field independence or field dependence.

### Online Shopping on the AR Platform

Users’ mental workload and SA are the keys to the supervisory control of safety–critical systems (Yang et al., 2012). Similarly, the mental workload and SA are the keys to AR online shopping.

The AR shopping platform should pay attention to psychological differences, physical differences and gender differences. The shopping platform should include three aspects of improvement, namely gender difference, personality difference and product value features. Male and female shopping platforms should highlight these differences. The mental workload of users of different genders was not the same. The marketing of differently gendered products should employ a diversified strategy. Through shopping data, we can establish a shopper’s personality model. Based on the shopper’s personality model, the retailer could make personalized APP experiences. The study suggested that AR shopping should try to use visual advertising.

### Research Limitations and Future Studies

Although most of our findings support our hypotheses, other features of the shopping experience may have influenced the results. One factor may be the participant selection. Because household surveys are difficult, the number of participants was small. The sample size can be further expanded. Second, the choice of just one item (the sofa) as high-priced item and of just one item (the mirror) as a low-priced item is a limitation. The choice of the item may be one of the influencing factors. The third factor is the contention in cognitive neuroscience. The study results should be verified through neuromarketing methods. Wireless wearable physiological equipment is available for future research. Fourth, AR online shopping will become the mainstream in future studies.

## Conclusion

In online shopping, the males’ mental workload was greater than the females’ mental workload. The property of high value was the main influencing factor for the high mental workload of the males. The psychological effect of AR technology and VR technology differs in online shopping. The method of online shopping, sensory channels and cognitive style interacted in online shopping.

## Ethics Statement

The study was approved by the academic and ethics committee of school of education in Hebei University. The participants who were intend to purchase furniture or visited in the furniture exhibition hall participated the experiment. The participants were asked finished their task in participants’ apartment. All participants provided their written informed consent to participate in this study.

## Author Contributions

Conceived and designed the study: XZ, CS, and XY. Performed the study: XZ and CS. Analyzed the data: XZ, CS, and CZ. Wrote the paper: XZ, CS, XY, and CZ.

## Conflict of Interest Statement

The authors declare that the research was conducted in the absence of any commercial or financial relationships that could be construed as a potential conflict of interest.
